# Machine Learning and Deep Learning Methods for Skin Lesion Classification and Diagnosis: A Systematic Review

**DOI:** 10.3390/diagnostics11081390

**Published:** 2021-07-31

**Authors:** Mohamed A. Kassem, Khalid M. Hosny, Robertas Damaševičius, Mohamed Meselhy Eltoukhy

**Affiliations:** 1 Department of Robotics and Intelligent Machines, Faculty of Artificial Intelligence, Kaferelshiekh University, Kaferelshiekh 33511, Egypt; cs.engineer.mohamed.1987@gmail.com; 2 Department of Information Technology, Faculty of Computers and Informatics, Zagazig University, Zagazig 44519, Egypt; 3 Department of Applied Informatics, Vytautas Magnus University, 44404 Kaunas, Lithuania; 4 Computer Science Department, Faculty of Computers and Informatics, Suez Canal University, Ismailia 41522, Egypt; mohamed_eltokhy@ci.suez.edu.eg

**Keywords:** skin image segmentation, skin lesion classification, machine learning, deep learning, small data, racial bias

## Abstract

Computer-aided systems for skin lesion diagnosis is a growing area of research. Recently, researchers have shown an increasing interest in developing computer-aided diagnosis systems. This paper aims to review, synthesize and evaluate the quality of evidence for the diagnostic accuracy of computer-aided systems. This study discusses the papers published in the last five years in ScienceDirect, IEEE, and SpringerLink databases. It includes 53 articles using traditional machine learning methods and 49 articles using deep learning methods. The studies are compared based on their contributions, the methods used and the achieved results. The work identified the main challenges of evaluating skin lesion segmentation and classification methods such as small datasets, ad hoc image selection and racial bias.

## 1. Introduction

The annual incidence of melanoma cases has increased by 53%. This is due in part to increased ultraviolet (UV) exposure [1]. Despite the fact that melanoma is one of the deadliest types of skin cancer, early identification can lead to a high chance of survival.

Cancer develops when cells in the body begin to proliferate uncontrollably. Metastasizing means that cancerous cells may form in practically any place of the body and spread [2]. In this regard, the uncontrolled proliferation of abnormal skin cells is referred to as skin cancer. Uncorrected DNA damage to skin cells, most typically produced by UV radiation from the sun or tanning beds, creates mutations, or genetic flaws, that cause skin cells to reproduce rapidly and produce malignant tumors.

**Figure 1:**
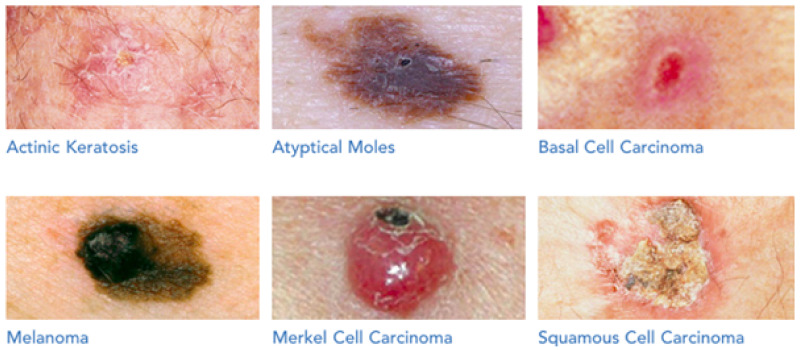
Different kinds of skin cancer classified by the Skin Cancer Foundation [4].

There are several varieties of benign and malignant melanomas that make the diagnosis of skin lesions complex. Squamous Cell Carcinoma (SCC), Basal Cell Carcinoma (BSC), and melanoma are major forms of irregular skin cells seen in clinical practice [3]. Further, the Skin Cancer Foundation (SCF) [4] distinguishes three less common types of abnormal cells, namely Merkel cell carcinoma, Actinic Keratosis (AKIEC), and Atypical moles. The six forms of skin lesions are depicted in Figure 1. The second most harmful cells are atypical moles after melanoma cases. According to SCF [4], the following are the distinctions between abnormal tissues:

1. Actinic Keratosis (AKIEC) or solar keratosis: This is a form of keratosis that occurs on the skin. It is a crusty, scaly growth on the skin. It is classified as pre-cancer because it has the potential to turn into skin cancer if left untreated.
2. Atypical moles: Also known as dysplastic nevi, they are benign moles that have an irregular appearance. They may look like melanoma, and the ones that have them are more likely to develop melanoma in a mole or anywhere on the body. They have a greater chance of having melanom;
3. Basal Cell Carcinoma (BCC): This is the most common form of skin cancer. This form of skin cancer spreads rarely. Its common symptoms are open sores, shiny bumps, red spots, pink growths, or scars;
4. Melanoma: This is the most lethal kind of skin cancer. It is often black or brown, although it can also be pink, red, purple, blue, or white. UV radiation from the sun or tanning beds causes cancerous tumors. Melanoma is frequently treatable if detected and treated early, but, if not, the disease can spread to other places of the body and the therapy would be complicated and deadly;
5. Merkel cell carcinoma: This is an uncommon and aggressive kind of skin cancer that has a high chance of metastasizing. However, it is 40 times less prevalent than melanoma;
6. Squamous Cell Carcinoma (SCC): This is the second most frequent kind of skin cancer. Scaly red spots, open sores, raised growths with a central depression, or warts are frequent signs.

The dominating cause of such skin cancer forms is skin tissue damage caused by UV radiation [5,6,7,8,9,10,11,12,13,14,15,16,17,18,19,20,21]. A dermatologist’s visual examination is a common clinical procedure for melanoma diagnosis [18]. The precision of the clinical diagnosis is somewhat deceptive [19]. Dermoscopy is a non-invasive diagnostic method that interconnects clinical dermatology with dermatology by enabling the display of morphological characteristics which are not discernible using a naked eye examination. The morphological details visualized can significantly be improved with different techniques such as solar scans [20], microscopy of the Epiluminescence (ELM), Cross-polarization epiluminescence (XLM), and side transillumination [21,22,23,24]. Therefore, the dermatologist receives further diagnostic criteria. Dermoscopy improves diagnostic performance by 10–30% relative to a non-discrete eye [25,26,27,28]. Nevertheless, [29,30,31] reported that the diagnostic accuracy of the dermoscopy was decreased with novice dermatologists in contrast with expert dermatologists because this process requires a great deal of experience to identify lesions [32].

According to ref. [33] professional dermatologists have achieved 90% sensitivity and 59% specificity in the identification of skin lesions. Around the same time, the statistics for less qualified doctors indicated a significant decline for general practitioners of about 62–63%.

A visual inspection by a dermatologist of the suspicious skin region is the first step in diagnosing a malignant lesion. A correct diagnosis is essential because certain types of lesions have similarities; moreover, the accuracy of the Computer-Aided System (CAD) is close to the experienced dermatologist’s diagnosis [34,35,36]. Without the use of technology, dermatologists can diagnose melanoma with a 65–80% accuracy rate [37]. For suspect cases, a dermatoscopic image is taken using a very high-resolution camera to complete the visual examination. The lighting is controlled and a filter is used during the recording to reduce skin reflections and thus visualize the deeper layers of skin. This technical assistance led to a 49% improvement in skin lesion diagnosis [31]. Ultimately, the combination of a visual examination and dermatoscopy images led to an absolute accuracy of 75–84% for melanoma detection [38,39].

Classifying lesions of the skin has been an aim of the machine learning community for some time. Automated classification of lesions is used in clinical examination to help physicians and allow rapid and affordable access to lifesaving diagnoses [40], and outside of the hospital environment, smartphone apps have been used [41]. Before 2016, most research adopted the traditional machine learning workflow of preprocessing (enhancement), segmentation, feature extraction, and classification [41,42,43]. These phases are explained in the following section:

1. Image enhancement: This phase aims to eliminate all noise and artifacts such as hair and blood vessels in dermoscopic images;
2. Segmentation: Segmenting the Region of Interest (ROI) is a crucial step in CAD systems. The process of segmenting skin cancer images is made more complex by a large number of different skin lesions. It quickly became one of the most complex and tedious tasks in the CAD system;
3. Feature extraction: After defining the ROI, the goal of the feature extraction step is to identify the best set of features that have high discrimination capability to classify the dataset into two or more classes;
4. Classification and detection: The proposed system is evaluated according to its capability to classify the dataset into different classes. Hence, the choice of classifier is critical for a better performance. However, it depends on the set of extracted feature and the required number of classes. The classification performance measures are accuracy, specificity, sensitivity, precision, and Receiver Operating Characteristic (ROC).

The need for high-performance CAD systems is essential for lesion detection and diagnosis. Feature selection is a crucial task for CAD system development. The choice of appropriate features took a long time for the automatic recognition of pigmented skin lesion images in 1987 [44]. In the same manner, errors and data loss have a significant influence on the classification rate. For example, an inaccurate segmentation result often results in poor outcomes in feature extraction and, thus, low accuracy in the classification. Machine vision and computer analysis are becoming more critical to produce a successful automatic melanoma diagnosing system [45,46,47,48,49,50]. An accurate CAD system would help doctors and dermatologists to make better and more dependable diagnoses.

Many CAD systems have been identified using different border detection, extraction, selection, and classification algorithms. Some studies [51,52,53,54,55,56,57,58] have proposed the study and analysis of image processing techniques to diagnose skin cancer; moreover, they compared Artificial Intelligence (AI) and CAD system performances against the diagnostic accuracy of experienced dermatologists. However, further work is required to define and reduce ambiguity in automated decision support systems to enhance diagnostic accuracy. There is no comprehensive and up-to-date review of the automatic skin lesion diagnostic model. The constant development, in recent years, of new dermoscopic research classification algorithms and techniques would benefit from such a study.

## 2. Methods

### 2.1. Systematic Review

We have looked for systematic reviews and original research papers written in English in the ScienceDirect, IEEE, and SpringerLink databases. In this analysis, only papers that were published in journals and recorded proper scientific proceedings were considered.

Papers were included based on the inclusion criteria: (i) classification or segmentation of skin lesions binary or multi-class, (ii) traditional machine learning method, (iii) deep learning models, (iv) digital image modality, (v) papers published in well-defined journals, and (vi) published in English.

The exclusion criteria were used to exclude the irrelevant studies based on the list of criteria presented as follows: (i) review articles, (ii) papers published in a language different from English, (iii) conference papers, (iv) books, and (v) book chapters.

**Figure 2:**
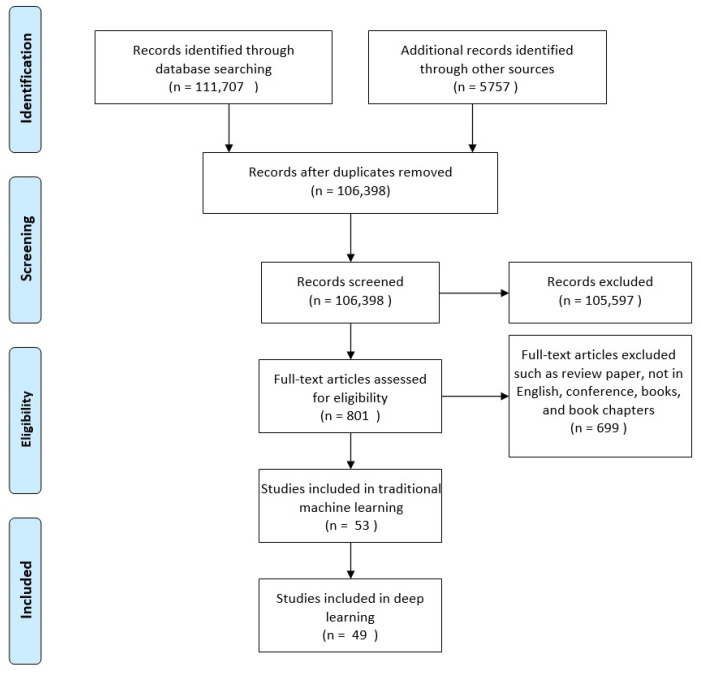
Study selection using PRISMA flow diagram.

The PRISMA flow diagram in Figure 2 shows the selection procedure [59]. The initial search identified 111,701 literature sources satisfying the search criteria. These sources were supplemented with 5757 records identified using other methods (forward and backward snowballing). After the removal of duplicate records, the number of papers ended up with 106,398 records. After applying the inclusion criteria, 801 full-text articles were identified, which were further inspected by applying the exclusion criteria. Finally, 53 articles using traditional machine learning methods and 49 articles using deep learning were selected. The selected articles were further analyzed and their results are discussed in this study. In addition, we listed only the models with the best score in each sample from studies that tested several models.

### 2.2. Datasets

The performance of melanoma diagnosis has improved with the dermoscopic method [21]. Dermoscopy is an invasive skin imaging technique that can capture enlightened and enlarged pictures of skin lesions to improve the clarity of the spots. The effect of the deeper skin could be improved by removing the reflection from the skin surface [60]. Automatic identification of dermoscopic images of melanoma is a challenging task due to many factors: firstly, intraclass variability in lesions such as texture, scale, and color; secondly, the high resemblance between the lesions of melanoma and non-melanoma; finally, the environmental conditions around it including hair, veins, and color calibration charts and rule marks.

In this section, the most used datasets in this area of research are described. A broad range of available and free online datasets such as MedNode, DermaIS, DermQuest, the ISIC 2016, 2017, 2018, and 2019, Ph2, and paid like Dermofit were used.

MED-NODE consists of 170 images of melanoma and nevus; it is divided into 70 and 100, respectively. The dataset came from the Digital Image Archive of the University of Medicine in the Netherlands, Department of Dermatology. The device for the sensing of skin cancer on microscopic images was developed and tested [61]. DermIS [62] is the Dermatology Information System skin dataset. This dataset is divided into two classes, nevus and melanoma. It contained 69 images: 26 nevus and 43 melanoma. DermQuest is a dataset consisting of 137 images. These images are divided into two classes, melanoma and nevus; these classes have 76 and 61 images, respectively [63].

The PH2 [64] database was created in collaboration with Porto University, Technical University of Lisbon, and Hospital Pedro Hispano in Matosinhos. It is comprised of 200 RGB color images with a 768 × 560 pixel resolution. This dataset has three groups of images: melanoma, normal nevus, and atypical nevus, with 40, 80, and 80 images in each category, respectively.

**Figure 3:**
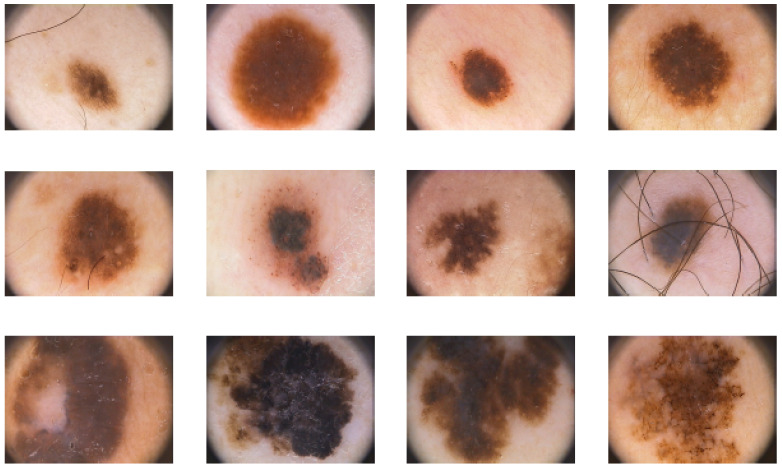
A graphic collection of images from the PH2 database, including common nevi (1st row), atypical nevi (2nd row), and melanomas (3rd row), https://www.fc.up.pt/addi/ph2%20database.html, accessed on 15 April 2021.

The skin colors characterized in the PH2 database may differ from white to creamy white. As illustrated in Figure 3, the images were carefully selected, taking into account their resolutions, quality, and dermoscopic features.

The International Skin Imaging Collaboration (ISIC) 2016 [65] dataset, which is referred to as the 2016 ISIC-ISBI challenge, provides 900 training images. Participants can produce and submit automated results using a separate test dataset (350 images). The training dataset consists of two classes. These classes are melanoma and benign, in which each class contains 173 and 727 dermoscopic images, respectively.

**Figure 4:**
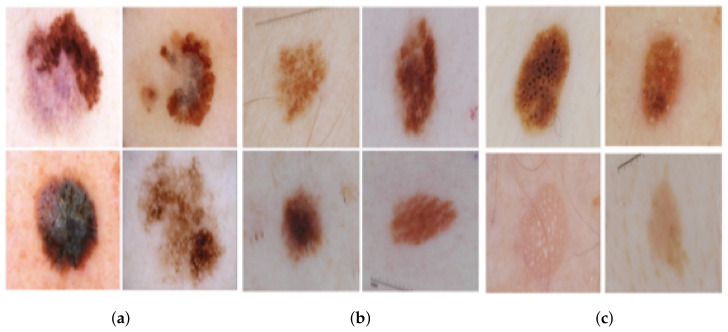
Different skin lesions examples of ISIC 2017: (**a**) melanoma, (**b**) nevus, and (**c**) seborrheic keratosis.

The International Skin Imaging Collaboration (ISIC) 2017 dataset [66], which is also referred to as the 2017 ISBI Challenge on Skin Lesion Analysis Towards Melanoma Detection. This challenge provides training data (2000 images), a separate validation dataset (150 photos), and a blind held-out test dataset (600 images). The training dataset consists of three classes divided into 374, 254, and 1372 dermoscopic images for melanoma, seborrheic keratosis, and nevus. Figure 4 shows different skin lesions examples from ISIC 2017.

The International Skin Imaging Collaboration (ISIC) 2018 dataset [67,68], which is also referred to as the HAM10000 (“Human Against Machine with 10,000 training images”) dataset, was divided into a training dataset, consisting of 10015 images, and the test dataset, consisting of 1512 images. This dataset was compiled using a variety of dermatoscopy techniques on all anatomic sites (except mucosa and nails) from a retrospective sample of patients who had undergone skin cancer screening at multiple institutions. The training dataset consists of seven classes: AKIEC, BCC, Benign Keratosis (BKL), Dermatofibroma (DF), Melanocytic nevus (NV), Melanoma (MEL), Vascular lesion (VASC). There are varying numbers of images in each of these groups. The MEL has 1113, the NV has 6705, the BCC has 514, the AKIEC has 327, the BKL has 1099, the DF has 115, and the VASC has 142. The classification of different images into seven groups in this dataset is one of the most difficult challenges.

**Figure 5:**
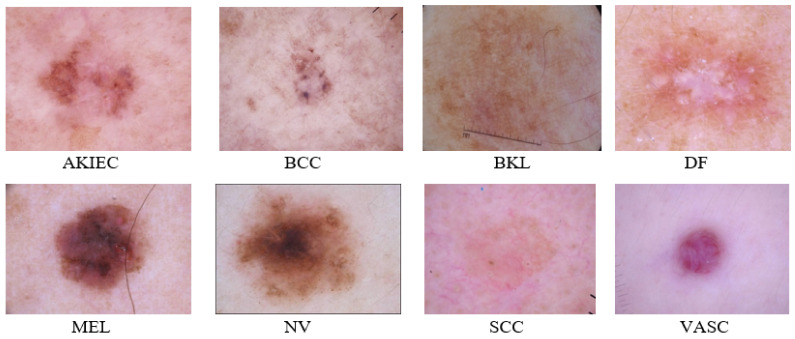
ISIC 2019 different skin lesions examples.

There is another dataset from the International Skin Imaging Collaboration, ISIC 2019 (BCN_20000) [69] consists of eight known classes and one class for outlier images. These classes are MEL, NV, BCC, AKIEC, BKL, DF, VASC, and SCC. ISIC 2019 consists of 25,331 images, where AKIEC has 867, BKL has 2624, BCC has 3323, DF has 239, NV has 12,875, MEL has 4522, SCC has 628, and VASC has 253. Figure 5 depicts the several types of skin cancer. This dataset is one of the hardest to categorize into eight classes with an uneven amount of photos in each class. The hardest challenge is to detect outliers or any of the other “out of distribution” diagnosis confidence.

The Dermofit Image Library dataset is composed of 1300 skin images with corresponding class labels and lesion segmentations. There are ten lesion categories in this dataset: AKIES, BCC, hemangioma, intraepithelial carcinoma, nevus, SCC, pyogenic granuloma, seborrhoeic keratosis, DF, and MEL. These classes have 331, 76, 257, 239, 65, 45, 97, 88, 78, and 24 images for nevus, MEL, seborrhoeic keratosis, BCC, DF, AKIES, hemangioma, SCC, Intraepithelial Carcinoma, and Pyogenic Granuloma [70].

The Interactive Atlas of Dermoscopy (EDRA) [71] is another type of skin image dataset that consisted of 20 labels. These labels named melanoma with several subtypes, BCC, blue nevus, Clark’s nevus, combined nevus, congenital nevus, dermal nevus, DF, lentigo, melanosis, recurrent nevus, Reed nevus, SK, and VASC.

The ISIC challenge 2020 dataset consisted of 33,126 dermoscopic images. These images were acquired from over 2000 patients. Images in the dataset were decomposed into nine classes in addition to an unknown image class [72]. Table 1 summarizes the total number of images and the total number of images in each class for all of these datasets.

For more than 30 years, skin cancer detection by CAD systems has been a hot topic of research [73]. For example, several methods for melanoma identification, classification, and segmentation have been developed and tested [74,75,76,77,78,79,80,81,82,83,84,85,86,87,88,89,90,91,92,93,94,95,96,97,98,99]. The following section addresses the researcher’s effort in the state of the art using journal papers published in ScienceDirect, IEEE, and SpringerLink databases during the last five years only.

### 2.3. Traditional Machine Learning

The rule asymmetry, border, color, diameter (ABCD) was used by authors in [100] to analyze the border and color of skin lesions. They classified the features using a multilayer perceptron network (MLP) based on backpropagation training. In [101], a Gabor filter and geodesic active contours are used to enhance the image and remove hair. Then, the ABCD scoring method was used to extract features. Finally, a combination of existing methods was used to classify lesions. In [102], the authors classified melanoma based on the thickness of the lesion by three values. Two classification schemata were used: the first classified lesions into thin or thick and the second schema classified lesions into thin, intermediate, and thick. They combined logistic regression with artificial neural networks for classification. In [103], lesions were enhanced using a median filter separately on each channel of the RGB space. Finally, these lesions were segmented based on a deformable model. A segmentation method based on the Chan–Vese model was proposed by [104]. Images were first enhanced using an isotropic diffusion filter, and then ABCD was used to extract the features from the segmented regions. These features were classified using a support vector machine (SVM). In [105], the authors proposed a classification system for BCC and MEL using Paraconsistent logic (PL) and annotation with two values. They extracted the degrees of evidence, formation pattern, and diagnosis comparison. The spectra values that were used to differentiate between normal, BCC and MEL were 30, 96, and 19, respectively. A Delaunay Triangulation was used in [106] to extract the binary mask of ROIs. The authors of [107] segmented the histopathological images by extracting the granular layer boundary, and then the intensity profiles were used to classify two lesions only.

A comparison of 4 classification methods that were used for skin lesion diagnosis is summarized in [108] to determine the best method. Based on the histopathology of BCC, the authors of [109] combined two or three Fourier transform features to form one Z-transform feature. A CAD system using principal component analysis (PCA) and SVM was proposed in [110] to classify skin psoriasis. A framework for the segmentation of BCC was proposed in [111]. The hemoglobin component was clustered using k-means. In [112], the classification system for skin cancer was based on deep lesions using 3D reconstruction. They utilized adaptive snake, stereo vision, structure from motion, depth from focus for segmentation, and classification.

In [113], the lesion was extracted using a self-generated neural network. Then, the descriptive border, texture, and color features were removed. Finally, an ensemble classifier network that combined fuzzy neural networks with backpropagation (BP) neural network was used to classify the lesions based on the extracted features. A fixed grid wavelet network was used by [114] to enhance and segment skin lesions. Then, these features were classified by D-optimality orthogonal matching pursuit. Based on a chaotic time series analysis of the boundary, Khodadadi et al. [115] analyzed the irregular boundary of an infected skin lesion by Lyapunov exponent and Kolmogorov–Sinai entropy.

In [116], a segmentation technique for skin lesions was proposed based on ant colony using three types of features from lesions such as texture, relative colors, and geometrical properties. Finally, these features were classified by two classifiers: artificial neural network (ANN) and K-nearest neighbour (KNN). Based on shape and color, the authors of [117] combined some features after segmentation using ABCD. Finally, these features were classified and tested individually and after being connected. The Histogram of Gradients (HOG) and the Histogram of Lines (HL) were used in [118] to create a bag of features for each one separately. The bag of features was used to extract texture and color features for skin lesion detection. Color features were extracted using 3rd Zernike moments.

Roberta et al. [119] proposed a skin lesion diagnosis based on the ensemble model for feature manipulation. Przystalski et al. [120] proposed multispectral lesion analysis by fractal methods. Jaisakthi et al. [121] proposed a segmentation method for skin lesions using the Grab-Cut algorithm and k-means. Do et al. [122] introduced a melanoma detection system using a smartphone. Images were acquired using a smartphone camera. Then, they searched for the best processing method that worked appropriately with smartphones. This method started with a hierarchical segmentation approach and numerical features to classify a skin lesion. Adjed et al. [123] proposed a feature extraction method using wavelet transform, curvelet transforms, and local binary pattern (LBP). Finally, the extracted features were classified using an SVM. Hosseinzade [124] divided lesion images based on fabric characteristics. Fabric characteristics were described by the Gabor filter, and then these characteristics were classified by k-means.

Akram et al. [125] proposed an automatic skin lesion segmentation and classification system. They used several diverse ABCD, fuzzy C-means, pair threshold binary decomposition, HOG, and linear discriminant analysis (LDA). Jamil et al. [126] proposed a technique for skin lesion detection. Finally, they classified lesions based on color, shape, Gabor wavelet, and Gray intensity features. Khan et al. [127] combined Bhattacharyya distance and variance as an entropy-based method for skin lesion detection and classification.

Tan et al. [128] enhanced Particle Swarm Optimization (PSO) for skin lesion feature optimization. The authors modified two PSO models for discriminative feature selection; the first one performing a global search by combining lesion features and an in-depth local search by separating lesions into specific areas. The second modified PSO was for random acceleration coefficients. Subsequently, these features were classified using different classification methods. Tajeddin et al. [129] classified skin melanoma based on highly discriminative features. They started with contour propagation for lesion segmentation. Based on the peripheral area to extract features, lesions were mapped by log-polar space using Daugman’s transformation. Finally, different classifiers were compared.

To classify skin lesions, the authors in [130] utilized the structural co-occurrence Matrix of frequencies extracted from dermoscopic images. Peñaranda et al. [131] classified skin lesions by analyzing skin cells using Fourier transform infrared. Finally, a study was conducted that used the perturbations that influenced results to determine the right effects. Wahba et al. [132] proposed a skin lesion classification system based on the Gray-level difference method. They tried to discriminate between four lesions by extracting features using ABCD and cumulative level-difference mean. Finally, these features were classified using an SVM. Zakeri et al. [133] proposed a CAD system to differentiate between melanoma and dysplastic lesions. They enhanced the grey-level co-occurrence matrix to extract features. Finally, these features were classified using an SVM. Pathan et al. [134] proposed a detection system for pigment networks and differentiated between typical and atypical network patterns. In [135], laser-induced breakdown spectroscopy was used with a combination of statistical methods to distinguish between the soft tissue of the skin.

Chatterjee et al. [136] utilized the non-invasive image of a skin lesion to distinguish melanoma from nevi. They obtained the texture pattern of the skin using 2D wavelet packet decomposition. Qasim et al. [137] proposed a skin lesion classification system. KNN was used with the enhanced images by the Gaussian filter to extract ROI. Finally, the segmented ROI was classified using an SVM. Madooei et al. [138] utilized a blue-whitish structure to differentiate melanoma from nevi lesions. Saez et al. [139] utilized the color of lesions to classify these lesions as melanoma or nevi. The lesions were classified by the color itself and their neighborhood color values. Navarro et al. [140] proposed a segmentation system for skin lesions. They classified the segmented lesions into melanoma and nevi. Riaz et al. [141] proposed a CAD system for skin lesions. Their system started with lesion segmentation to extract ROI. They utilized Kullback–Leibler divergence to detect lesion boundaries.

Sabbaghi et al. [142] presented a QuadTree based on the perception of lesion color. They found that the three most common colors of melanoma were blue-grey, black, and pink. Finally, they used different classifiers, such as SVM, ANN, LDA, and random forests (RFs). Murugan et al. [143] utilized watershed segmentation to extract ROI. Features were extracted using ABCD and Gray-Level Co-occurrence Matrix (GLCM). Finally, these features were classified using KNN, RF, SVM. Khalid et al. [144] suggested a segmentation method for dermoscopic skin lesion images using a combination of wavelet transform with morphological operations. Majumder et al. [145] proposed three features that were used in melanoma classification based on the ABCD rule. Chatterjee et al. [146] introduced fractal-based regional texture analysis (FRTA) to extract shape, fractal dimension, texture, and color features to classify three lesions using SVM with RBF.

Chatterjee et al. [147] proposed a classification system for four kinds of lesions. Features were extracted using cross-correlation techniques based on frequency domain analysis. Their system differentiated between benign and malignant lesions of the epidermal and melanocytic classes in a binary manner. Upadhyay et al. [148] extracted color, orienttion histogram, and gradient location of skin lesion features. These features were fused and classified as benign or malignant using an SVM. Pathana et al. [149] proposed a skin lesion CAD system. Garcia-Arroyo et al. [150] proposed a skin lesion detection system. Lesions were segmented using fuzzy histogram thresholding. Hu et al. [151] suggested a skin lesion classification approach. To measure the similarity between features, they introduced codewords for a bag of features. Moradi et al. [152] suggested a skin lesion segmentation and classification system based on sparse kernel representation. Pereira et al. [153] proposed a skin lesion classification system based on characteristics of lesions borderline in addition to combining LBP with gradients.

### 2.4. Deep Learning

Kawahara et al. [154] proposed a skin lesion classification system using a convolutional neural network (CNN). The modified pre-trained CNN was able to classify images with different resolutions. Yu et al. [155] proposed a novel CNN for skin lesion segmentation and classification. The proposed CNN was based on residual learning and consisted of 50 layers. Codella et al. [156] proposed a deep residual network for skin lesion classification using the benchmark dataset ISIC2016. Bozorgtabar et al. [157] proposed a decision support system that localized skin cancer automatically using deep convolution learning for pixel-wise image segmentation. Yuan et al. [158] proposed a skin lesion segmentation system by leveraging CNN. The network consisted of 19 layers. Instead of using the traditional loss function, they utilized Jaccard Distance as a loss function.

Sultana et al. [159] proposed a skin lesion detection system using deep residual learning with a regularized framework. Rundo et al. [160] utilized ABCD for lesions to analyze skin lesions. Finally, ad hoc clustering was performed using a pre-trained deep Levenberg–Marquardt neural network. Creswell et al. [161] proposed denoising adversarial autoencoders to classify limited and imbalanced skin lesion images. Harangi [162] ensembled four different CNNs to investigate the impact on performance. Guo et al. [163] utilized and ensembled multi-ResNet to analyze skin lesions. The training images for each ResNet were pre-treated in different ways while the labels were still like the original.

Monedero et al. [164] utilized the thickness of lesions to detect melanoma using the Breslow index. The extracted texture, shape, pigment network, and color features of lesions were classified using GoogleNet to classify lesions into five types. Hagerty et al. [165] utilized deep learning and conventional image processing to extract different skin lesion features. The extracted features from deep learning and traditional processing of images were combined and fused. Finally, the newly generated features were used to classify lesions. Polap [166] proposed a skin lesion classification based on IoT. He used deep learning over IoT. The proposed model classified images in a short time, but with a low classification rate. Such a system could be used in a smart home as a part of an intelligent monitoring system [167].

Sarkar et al. [168] proposed a depth-wise separable residual deep convolutional network to classify skin cancer. The non-local means filter was succeeded by the contrast-limited adaptive histogram equalization (CLAHE) over the discrete wavelet transform (DWT) algorithm. Zhang et al. [169] proposed a CNN model with attention residual learning to classify skin lesions into three classes. The proposed deep model has four residual blocks with a total of 50 layers that consist of the deep model. Albahar [170] introduced a skin lesion detection system for binary classification of malignant or benign. He proposed a CNN model consisting of seven layers. He also proposed a regularization method to control the complexity of the classifier using the standard deviation of the weight matrix.

González-Díaz [171] introduced a skin lesion CAD system using CNN called DermaKNet. The proposed CNN was based on ResNet50, but the author started by applying a modulation block over the convolutional res5c layer outputs. Two pooling layers (AVG and Polar AVG) were worked together at the same time. Three fully connected layers were used at the end of CNN. However, because melanoma growth differently, the third fully connected one precedes the asymmetry block. The asymmetry block was used to detect the different methods of melanoma growth.

Kawahara et al. [172] proposed a skin lesion classification system using CNN that simultaneously worked on multiple tasks. The proposed CNN is able to classify seven-point melanoma checklist criteria. The proposed CNN skin lesion diagnosis classified skin lesion images and meta-data of patients. Yu et al. [173] proposed a skin lesion classification system using CNN and the local descriptor encoding method. The lesion features were extracted from images using ResNet50 and ResNet101. Then, a fisher vector (FV) was used to build a global image representation using the extracted features of ResNet. Finally, an SVM was used with a Chi-squared kernel for classification. Dorj et al. [174] proposed a skin cancer classification system using CNN. The pre-trained Alex-Net was used to extract features while the Error-Correcting Output Codes (ECOC) SVM was used to classify the extracted features.

Gavrilov et al. [175] proposed a skin neoplasm (cancer) classification system using CNN. They applied transfer learning to inception V3 (Googlenet). Furthermore, the development of web and mobile applications was created to allow patients to assess their lesions and give a preliminary diagnosis using images captured by themselves. Chen et al. [176] proposed a classification system for facial skin diseases. They used three CNN models with transfer learning to classify five skin diseases of the face. The proposed model was worked on through a cloud platform. Mahbod et al. [177] utilized four different CNN models (AlexNet, VGG, ResNet-18, and ResNet-101) to classify three skin lesions. They used SVM, RF, and MLP to classify the extracted features from CNN. The different classification results were enameled together to generate a single classification for the input lesions. Brinker et al. [178] proposed a skin lesion classification system using a pre-trained model. The modified pre-trained model ResNet50 outperformed expert dermatologists in classifying lesions into melanoma and nevus.

Tan et al. [179] utilized PSO for skin lesion segmentation. They tried to optimize PSO using different methods, such as Firefly Algorithm (FA), spiral research action, probability distributions, crossover, and mutation. To enhance lesion segmentation, K-Means was used. The hybrid learning PSO (HLPSO) was used in the development of CNN. The classification system could classify lesions into melanoma and nevus. Khan et al. [180] used a custom CNN of ten layers for image segmentation and a deep pre-trained CNN model for feature extraction. Then, an improved moth flame optimization (IMFO) algorithm was used for feature selection. The selected features were fused using multiset maximum correlation analysis and classified using the Kernel Extreme Learning Machine (ELM) classifier.

Tschandl et al. [181] proposed combining and expanding different CNNs for the segmentation and classification of skin lesions. They used three well-known benchmark datasets. Finally, they found that post-processing with a small dataset that contains noise decreased Jaccard loss. Vasconcelos et al. [182] proposed a skin lesion segmentation system using morphological geodesic active contour. Different CNN models were used, such as full resolution convolutional networks (FrCNs), deep class-specific learning with probability-based step-wise integration (DCL-PSI). The proposed model was able to classify skin lesions into melanoma and nevus.

Burlina et al. [183] proposed a CNN for acute Lyme disease from erythema migrans images, even with different acquisition and quality conditions. They fine-tuned and replaced the final layers of ResNet50. The proposed model was able to classify four different lesions. Maron et al. [184] proposed a system that classified five types of skin lesions. They applied transfer learning to ResNet50 in addition to fine-tuning CNN weights. They compared the classification rate using a CNN against 112 expert dermatologists.

Goyal et al. [185] proposed a skin lesion segmentation system by ensembling the segmentation output from Mask R-CNN and DeeplabV3+. Albert [186] proposed Predict-Evaluate-Correct K-fold (PECK), which trains CNNs from limited data. The proposed system was used for skin lesion classification in his research, Synthesis, and Convergence of Intermediate Decaying Omnigradients (SCIDOG), to detect the contour of lesions. Finally, the segmented lesions were classified using a CNN with SVM and RF. Ahmad et al. [187] utilized three loss functions by fine-tuning ResNet152 and InceptionResNet-V2 layers. Euclidean space was used to compute the L-2 distance between images. Finally, the L-2 distance was used to adapt the weights to classify images.

Kwasigroch et al. [188] proposed a skin lesion classification using a CNN with hill-climbing for search space. This process led to increasing the size of the network, which reduced the computational cost. Adegun et al. [189] proposed an encoder and decoder network with subnetworks connected using skip connections. The proposed CNN was used for skin lesion segmentation and pixel-wise classification. Song et al. [190] suggested that CNNs could segment, detect, and classify skin lesions. To control the imbalanced datasets, they utilized a loss function based on the Jaccard distance and the focal loss. Wei et al. [191] proposed a skin lesion recognition system based on fine-grained classification to discriminate features. A different lightweight CNN was utilized for segmentation and classification.

Gong et al. [192] proposed a dermoscopic skin image classification system using deep learning models. The authors enhanced skin images using StyleGANs while the enhanced image was classified using 43 CNNs. CNNs were divided into three groups with different fusion methods. Finally, the classification decision was generated using the max voting technique.

Nasiri et al. [193] proposed a skin lesion classification system based on deep learning with case-based reasoning (CBR). Öztürk et al. [194] proposed a segmentation system for skin lesions. Hosny et al. [195] proposed a new deep CNN classification system for skin lesions. The authors performed three different experiments with three datasets. They compared the accuracy between using traditional machine learning classifiers and the emerging technology with CNN. They found that the conventional machine learning classifier led to a lower classification rate.

Amin et al. [196] proposed a skin lesion classification system. Firstly, they enhanced images, then biorthogonal 2-D wavelet transforms and the Otsu algorithm were used to segment lesions. Finally, two pre-trained models were fused serially to extract features for classification using PCA. Mahbod et al. [197] proposed investigating the effect of the different image sizes of skin lesions using transfer learning with pre-trained models.

Hameed et al. [198] proposed a skin lesion classification system based on a multiclass multilevel algorithm. Traditional machine learning and deep learning methods were used with the proposed model. Zhang et al. [199] proposed an optimization algorithm for optimal weight selection to minimize the network output error for skin lesion classification. Hasan et al. [200] proposed a semantic segmentation network to segment skin lesions called DSNet. They utilized depth-wise separable convolution to reduce the number of parameters that produced a lightweight network. Al-masni et al. [201] proposed a diagnostic framework for skin lesion classification systems, which combined segmentation of lesion boundaries with multiple classification stages. The proposed system segmented the lesions using a full resolution convolutional network (FrCN) with four CNNs for classification. This system was evaluated and tested using three benchmark datasets.

**Figure 6:**
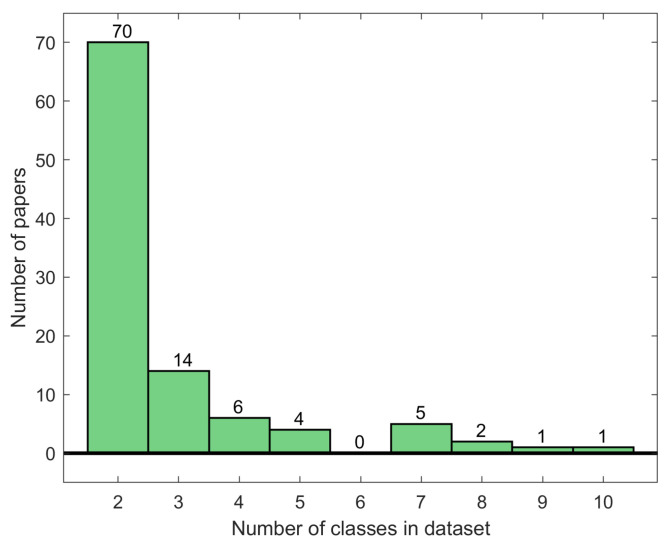
Number of classes used for skin disease recognition.

Pour et al. [202] proposed a segmentation method for skin lesions using a CNN. The CNN was trained from scratch using a small dataset with augmentation. Olusola et al. [203] utilized image augmentation (a variant of SMOTE). Then, they classified skin lesions into benign and malignant using SquuezeNet. Hosny et al. [204] utilized Alexnet with transfer learning to classify the challenging dataset ISIC2018. They used different approaches for lesion segmentation. Hosny et al. [205] proposed a CAD system for skin lesions using the challenging dataset ISIC2019. That dataset has several challenges, such as imbalanced classes and unknown images. The authors utilized the bootstrap weighted classifier with a multiclass SVM. This classifier changed the weights according to the image class. Finally, the authors dealt with unknown images in two different ways. They trained GoogleNet with a new class containing a different number of unknown images collected from various sources. The second way was the similarity score; if the high similarity score of the tested image with the known eight classes was less than 0.5, the tested image was identified as an unknown image or out of distribution. The number of classes used for skin disease recognition in the analyzed works is summarized in Figure 6. Generally, the vast majority of works used two-class recognition only, while only one study [172] used 10 classes for recognition.

## 3. Discussion and Conclusions

The paper is an analytical survey of the literature on skin lesion image classification and disease recognition. It is a comprehensive review of the methods and algorithms used in the processing, segmentation, and classification of skin lesion images. Both classical machine learning methods and deep convolutional neural network models were explored. A discussion of the available and known datasets with a comparison between these datasets was introduced. At the end of the systematic survey, a table was used to compare state-of-the-arts methods in a novel way. The column “simple” in Table 2 refers to the proposed method not being complicated and easy to apply and did not require specified hardware, whereas the column “Contribution Achieved” was added to indicate if the research paper achieved what was discussed or not.

The comparison of methods of classification for skin lesions shows that the problem formulations of each work vary slightly. The efficient melanoma detection process has five core elements, focused on data acquisition (collection), fine-tuning, selection of features, deep learning, and final model development. The first step is to acquire data in which data from publicly available benchmarks, non-listed and non-public databases, such as the melanoma detection images collected from the internet, are used for detecting skin cancer.

There were several methods of learning with regard to deep learning based on transfer learning, while others were based on ensemble approaches, and some employed neural networks and hybrid techniques of fully convolutional neural networks. The pre-trained deep learning models and handcrafted methods that were based on a deep-leaning approach have already shown promising results for high-precision accuracy of melanoma detection.

There is a limited range of images for training and testing available for comparison as most of the datasets are small. With small datasets, the proposed methods do well, but are prone to over-fitting, and when tested with a large image set, are reliably unpredictable. For example, just 200 images are included in the PH2 dataset. The problem of training with a small dataset could be mitigated by data augmentation, image generation by an adversarial generative network, and transfer learning. Some researchers use non-public databases and internet images. This makes it more difficult to replicate the findings and results because the dataset is not available, whereas the selection of images from the internet may be biased.

The creation of large public image datasets with images as representative of the world’s people as possible to avoid racial bias [206] is another major task in this research field. Image prejudice based on gender and race AI prejudice means that the models and algorithms fail to give optimal results for people of an under-represented gender or ethnicity.

Mostly, skin lesions from light-colored skin can be seen in current datasets. For example, the ISIC dataset images are mostly obtained in the USA, Europe and Australia. In addition, CNNs try to extract the skin color to achieve a proper classification for dark-skinned humans. This can only happen if the training dataset contains sufficient images of dark-skinned people. The size of the lesion also has significant importance. If the lesion size is smaller than 6mm, melanoma can not be detected easily.

The addition of clinical data such as race, age, gender, skin type, as inputs for classifiers may help to increase classification accuracy. This supplemental data could be beneficial for dermatologists’ decision making. These aspects should be included in future work. Finally, according to the authors’ perspective and based on the size of the dataset, if the dataset contains a large number of images per class, deep learning is better than traditional machine learning. Even with datasets containing few images, deep learning can overcome this issue by using different methods of augmentation. Deep learning makes intelligent decisions on its own with a higher accuracy rate. The pre-trained deep learning models and handcrafted methods that were based on a deep learning approach have already shown promising results for the high-precision accuracy of melanoma detection.

## Figures and Tables

**Table 1:** Summary of all the discussed datasets.

	Nevus or Atypical Nevus	Common Nevus	Melanoma	Seborrheic Keratosis	Basal Cell Carcinoma	Dermatofibroma	Actinic Keratosis	Vascular Lesion or Hemangioma	Squamous Cell Carcinoma	Intraepithelial Carcinoma	Pyogenic Granuloma	Total
MedNode	100	-	70	-	-	-	-	-	-	-	-	170
Dermis	26	-	43	-	-	-	-	-	-	-	-	69
DermQuest	61	-	76	-	-	-	-	-	-	-	-	137
Ph2	80	80	40		-	-	-	-	-	-	-	200
ISIC 2016	726	-	173	-	-	-	-	-	-	-	-	899
ISIC 2017	1372	-	374	254	-	-	-	-	-	-	-	2000
ISIC 2018	6705	-	1113	1099	514	115	327	142	-	-	-	10,015
ISIC 2019	12,875	-	4522	2624	3323	239	867	253	628			25,331
Dermofit	331	-	76	257	239	65	45	97	88	78	24	1300
EDRA	560	55	196	45	42	20	-	29	-	64	-	1011
ISIC2020	46	5193	584	135	7	-	37	-	-	-	-	6002

**Table 2:** A comparison between state-of-the-arts methods with novel methods.

Reference	Simple	Dataset			Colored Images	Enhancement	Segmentation	Contribution	Contribution Achieved	Methods and Tools	No. of Classes	Accuracy (%)	Sensitivity (%)	Specificity (%)	Precision (%)	Dice (%)
		Number	Type	Free												
[100]	Y	1	D	Y	N	Y	Y	Mobile Assessment	N	ABCD, MLP	2	88	66	93	N/A	N/A
[101]	N	1	A	N	N	Y	Y	Classify lesion Atlas	Y	Gabor filter, ABCD	2	94	91.25	95.83	N/A	N/A
[102]	N	1	A	N	Y	Y	Y	Classify lesion Atlas based on the thickness	Y	logistic regression with ANN	2	64.4	55.2	N/A	N/A	N/A
[103]	N	1	A	N	Y	Y	Y	Increase classification using a novel segmentation method	Y	Median Filter	2	N/A	N/A	N/A	N/A	N/A
		1	D	Y												
[104]	N	3	A	N	Y	Y	Y	Segment and classify pigmented lesions	Y	Anisotropic Diffusion, Chan–Vese’s, ABCD, SVM	2	79.01	N/A	N/A	80	N/A
		1	D	Y												
[105]	N	1	RS	N	-	-	-	classification	Y	Paraconsistent analysis network	3	93.79	N/A	N/A	N/A	N/A
[106]	Y	1	D	Y	N	Y	Y	Segmentation	Y	Delaunay Triangulation	2	75.91	67.46	95.93	N/A	N/A
[107]	N	1	H	N	Y	N	Y	Segmentation and Classification	Y	Granular Layer, Intensity Profiles	2	92.1	N/A	97.6	88.1	N/A
[108]	Y	1	F	N	-	-	-	Comparison of four Classification methods	Y	KNN with Sequential Scanning selection, KNN with GA, ANN with GA, Adaptive Neuro-Fuzzy Inference	2	94	N/A	N/A	N/A	N/A
[109]	Y	1	H	N	Y	N	Y	Classification lesions with and without segmentation	Y	Z-transforms	2	85.18	91.66	80	78.57	N/A
[110]	Y	1	JPGE	N	Y	N	N	Classification of Psoriasis	Y	PCA, SVM	2	100	100	100	N/A	N/A
[111]	Y	3	A	N	N	N	Y	Segmentation	Y	k-means	2	N/A	N/A	N/A	N/A	N/A
[112]	Y	1	A	N	Y	N	Y	Classification of skin cancer based on the deep of lesion using 3D reconstruction	Y	adaptive snake, stereo vision, structure from motion, depth from focus	8	86	98	99	N/A	N/A
		1	D	Y							3					
[113]	N	2	D	N	N	N	Y	Classification of melanoma	Y	a self-generated neural network, fuzzy neural networks with backpropagation (BP) neural networks	2	94.17	95	93.75	N/A	N/A
[114]	N	1	JPGE	N	Y	Y	Y	Segmentation and Classification	Y	fixed grid wavelet network, D-optimality orthogonal matching pursuit	2	91.82	92.61	91	N/A	N/A
[115]	N	1	SI	N	N	N	Y	Classification of the regular and irregular boundary of skin lesion	Y	1D time series for Lyapunov exponent and Kolmogorov–Sinai entropy	2	95	100	92.5	86.5	N/A
		1	D	Y												
[116]	Y	2	D	Y	Y	N	Y	Segmentation and classification of lesions to melanoma and Benign	Y	An ant colony, KNN, ANN	2	N/A	N/A	N/A	N/A	N/A
[117]	N	1	A	N	Y	N	Y	Segmentation and combine features, classification	Y	ABCD, SVM	2	90	72.5	94.4	N/A	N/A
		2	D	Y												
[118]	N	2	D	N	N	Y	Y	Codebook generated from a bag of features	Y	Histogram of Gradients, Histogram of Lines, Zernike moments	2	92.96	96.04	84.78	N/A	N/A
[119]	N	1	D	Y	Y	N	Y	Classification of skin lesion using feature subset selection	Y	Optimum-path forest integrated with a majority voting	2	94.3	91.8	96.7	N/A	N/A
[120]	N	1	SIA scope	N	N	N	Y	Analysis of multispectral skin lesion	Y	Box-counting dimension and lacunarity, Hunter score pattern detection, RBF kernel, SVM	2	97	59.6	97.8	N/A	N/A
[121]	N	2	D	Y	Y	Y	Y	Automatic segmentation of skin lesion	Y	The grab-Cut algorithm, k-means	2	96.04	89	98.79	N/A	91.39
[122]	Y	1	CCD	N	Y	N	Y	Classification of skin lesion using a smartphone	Y	Otsu’s, Minimum Spanning Tree, Color Triangle	2	87.98	89.09	86.87	N/A	N/A
[123]	Y	1	D	Y	Y	Y	N	Enhancement and fusion of skin lesion features for classification	Y	Wavelet transform, Curvelet transform, local binary pattern, SVM	2	86.17	78.93	93.25	N/A	N/A
[124]	N	-	-	-	N	Y	Y	Extract fabric characteristics for lesion classification	Y	Gabor filters based on shark smell optimizing method, K-means	-	N/A	N/A	N/A	N/A	N/A
[125]	N	3	D	Y	N	Y	Y	Skin lesion segmentation and recognition based on fused features	Y	ABCD, fuzzy C-means, pair threshold binary decomposition, HOG, linear discriminant analysis, linear regression, complex tree, W-KNN, Naive Bayes, ensemble boosted tree, ensemble subspace discriminant analysis	2, 3	99	98.5	99	98.5	N/A
[126]	N	1	D	Y	Y	Y	Y	Skin lesion segmentation with several classifiers	Y	2-D Gabor wavelet, OTSU’s, median filtering, morphological operations, m- mediod classifier, SVM, Gaussian Mixture Modeling	2	97.26	96.48	96.9	N/A	N/A
[127]	N	3	D	Y	Y	Y	Y	Classify the selected features of parallel fusion of features after segmentation	Y	Global maxima and minima, uniform and normal distribution, mean, mean deviation, HOG, Harlick method, M-SVM	2	93.2	93	97	93.5	N/A
[128]	N	3	D	Y/N	N	Y	Y	Optimize features of skin lesion and classify these features	Y	Particle Swarm Optimization, KNN, SVM, decision tree	2	N/A	N/A	N/A	N/A	N/A
[129]	N	1	D	Y	Y	Y	Y	Using high discriminative features for melanoma classification	Y	Histogram correction, OTSU’s, corner detection, Gray-level co-occurrence matrix features, Daugman’s rubber sheet model, RUSBoost, linear SVM	2	95	95	95	N/A	92
[130]	N	3	D	Y	N	Y	Y	Classify melanoma based on the main frequencies from dermoscopic images	Y	ABCD, HU Moments, GLCM, Structural Co-occurrence matrix, MLP, LSSVM, Minimal Learning Machine	2	89.93	92.15	89.9	N/A	91.05
[131]	N	1	I	N	-	Y	Y	Differentiate infrared spectroscopy of skin lesion	Y	Fourier transform infrared, Morphological information, PCA	2	85	N/A	N/A	N/A	N/A
[132]	N	1	D	Y	Y	Y	Y	Classifying four skin lesions based on the Gray-level difference method	N	Gray-level difference method, ABCD, SVM	4	100	100	100	N/A	N/A
[133]	N	1	CCD	N		Y	Y	Segment skin lesion and using a hybrid classifier to classify these lesions	Y	ABCD, pigment distribution and texture, GLCM, Log-linearized Gaussian mixture neural network, KNN, linear discriminant analysis, LDA, SVM, majority vote	3	98.5	95	99.5	N/A	N/A
[134]	N	1	D	Y	Y	Y	Y	Classify typical and atypical pigment network to diagnose melanoma	Y	Laplacian filter, median filter, polynomial curve fitting, connected component analysis, 2D Gabor filters, Gray-Level co-occurrence Matrix, Pearson product-moment co-relation, Probabilistic SVM, ANN	2	86.7	84.6	88.7	N/A	N/A
[135]	N	1	LIBS spectra	N	-	Y	Y	Classification of skin tissue	Y	laser-induced breakdown spectroscopy, PCA, KNN, SVM	2	76.84	74.2	86.9	N/A	N/A
[136]	Y	2	A	N	N	Y	Y	Classification of melanoma versus nevi by correlation bias reduction	Y	2D wavelet packet decomposition, SVM recursive feature elimination (SVM -RFE)	2	98.28	97.63	100	N/A	N/A
		2	D	Y												
[137]	Y	1	D	N	Y	Y	Y	Classification of lesions into melanoma and nevi	Y	Gaussian Filter, KNN, SVM	2	96	97	96	97	N/A
[138]	Y	1	A	N	Y	N	Y	Using of blue-whitish structure for melanoma classification	Y	multiple instance learning (MIL) paradigm, Markov network, SVM	2	84.5	74.42	87.9	61.54	N/A
		1	D	Y												
[139]	N	1	A	N	Y	N	Y	Classify lesion into melanoma or nevi based of color features	Y	K-means, pixel-based classification	2	89.42	N/A	N/A	N/A	N/A
[140]	Y	1	D	Y		Y	Y	Segmentation and classification of skin lesions	Y	Hough Transform, ABCD, SP-SIFT, LF-SLIC region labeling	2	96	N/A	N/A	N/A	93.8
[141]	Y	2	D	Y	Y	Y	Y	Detect the boundaries of lesions to classify into melanoma and nevi	Y	Kullback-Leibler divergence, local binary patterns, SVM, KNN	2	80.7	N/A	N/A	N/A	N/A
[142]	N	1	JPGE	N	Y	Y	Y	A QuadTree-based melanoma detection system based on color	Y	hybrid thresholding method, adaptive histogram thresholding, Euclidean distance transform, QuadTree, Kolmogorov-Smirnov, SVM, ANN, LDA, random forests	2	73.8	75.7	73.3	N/A	N/A
[143]	Y	1	D	Y	N	Y	Y	Segmentation and classification of skin lesion	Y	Median Filter, watershed segmentation, ABCD, GLCM, KNN, RF, SVM	2	89.43	91.15	87.71	N/A	N/A
[144]	Y	1	D	Y	N	Y	Y	Segmentations of Enhanced dermoscopic lesion images	Y	wavelet transform, morphological operations, Gray Thresholding, Cohen–Daubechies–Feauveau biorthogonal wavelet, Active contour, Color enhancement, Adaptive thresholding, Gradient vector flow	2	93.87	N/A	N/A	N/A	92.72
[145]	Y	1	D	Y	N	Y	Y	Three distinct features to classify melanoma	N	ABCD, Otsu, Chan–Vese, Dull-Razor, ANN	2	98.2	98	98.2	N/A	N/A
[146]	N	2	A	N	N	Y	Y	Classification three lesions based on shape, fractal dimension, texture, and color features	Y	recursive feature elimination, GLCM, fractal-based regional texture analysis, SVM, RBF	3	98.99	98.28	98.48	N/A	91.42
		2	D	Y												
[147]	N	2	A	N	Y	N	N	Extraction of features using frequency domain analysis and classify these features	Y	Cross spectrum-based feature extraction, Spatial feature extraction, SVM-RFE with CBR	4	98.72	98.89	98.83	N/A	N/A
		2	D	Y												
[148]	Y	1	D	N	Y	N	N	Improve bag of dense features to Classify skin lesions	Y	Gradient Location, Orientation Histogram, color features, SVM	2	78	N/A	N/A	N/A	N/A
[149]	N	2	D	Y	N	Y	Y	CAD system for clinical assist	N	chroma based deformable models, speed function, Chan-Vese, Wilcoxon Rank Sum statistics, Discrete Wavelet Transform, Asymmetry, and Compactness Index, SVM	2	88	95	82	N/A	N/A
[150]	Y	2	D	Y	N	Y	Y	Segmentation of skin lesions using fuzzy pixels classification and histogram thresholding	Y	fuzzy classification, histogram thresholding	2	88.4	86.9	92.3	N/A	76
[151]	N	1	D	Y	Y	Y	Y	Lesion classification based on feature similarity measurement for codebook learning in the bag-of-features model	Y	Codebook learning, k-means, color histogram, scale-invariant feature transform (SIFT)	2	82	80	83	N/A	N/A
		1	C	Y												
[152]	Y	1	D	Y	Y	Y	Y	Segmentation and classification of skin lesions using Kernel sparse representation	Y	Sparse coding, kernel dictionary, K-SVD	3	91.34	93.17	91.48	N/A	91.25
[153]	N	1	D	N	N	Y	Y	Improve skin lesion classification using borderline characteristics	Y	Gradient-based Histogram Thresholding, Local Binary Patterns Clustering, Euclidean distance, Discrete Fourier Transform spectrum (DCT), power spectral density (PSD), SVM, Feedforward Neural Network (FNN)	2	91	68	96	N/A	N/A
		1	C	Y												
[154]	Y	1	D	N	Y	N	Y	Multi-resolution-Tract CNN	N	AlexNet, GPU	10	79.5	N/A	N/A	N/A	N/A
[155]	Y	1	D	Y	Y	Y	Y	Automatic segmentation and classification for skin lesions	Y	CNN with 50 layers, residual learning, SoftMax, SVM, Augmentation, GPU	2	94	N/A	N/A	N/A	N/A
[156]	N	1	D	Y	N	Y	Y	Classification of segmented skin lesions	Y	U-Net, Sparse coding, Deep Residual Network (DRN), Augmentation	2	76	82	62	N/A	N/A
[157]	Y	1	D	Y	N	Y	Y	Segmentation of skin lesions using deep learning	Y	fully convolutional networks (FCN), VGG, Augmentation	2	N/A	N/A	N/A	N/A	89.2
[158]	Y	1	D	Y	Y	Y	Y	Automatic Skin Lesion Segmentation Using CNN	Y	FCN with 19 layers, Jaccard Distance, Augmentation, GPU	2	95.5	91.8	96.6	N/A	92.2
[159]	Y	3	D	Y	Y	Y	N	Detection of melanoma using CNN and regularized fisher framework	Y	ResNet50, transfer learning, SoftMax, SVM, Augmentation, GPU	2	78.3	35	88.8	N/A	N/A
		1	C	Y												
[160]	N	1	D	Y	N	Y	Y	Evaluation of skin lesion using Levenberg neural networks and stacked autoencoders clustering	Y	ABCD, morphological analysis, Levenberg–Marquardt neural network	2	N/A	98	98	N/A	N/A
[161]	N	1	D	Y	Y	N	N	Classify limited and imbalanced skin lesion images	N	Adversarial Autoencoder with 19 layers, Augmentation, GPU	2	N/A	N/A	83	N/A	N/A
[162]	Y	1	D	Y	Y	N	N	Ensemble different CNN for skin lesion classification	Y	GoogLeNet, AlexNet, ResNet, VGGNet, Sum of the probabilities, Product of the possibilities, Simple majority voting (SMV), Sum of the maximal probabilities (SMP), Weighted ensemble of CNN, Augmentation, GPU	3	86.6	55.6	78.5	N/A	N/A
[163]	N	1	D	Y	Y	N	N	Skin lesion analysis using Multichannel ResNet	Y	Ensemble multi-ResNet50, ANN, concatenated Fully Connected Layer, Augmentation, GPU	3	82.4	N/A	N/A	N/A	N/A
[164]	N	1	A	N	Y	Y	Y	Classify skin lesions based on border thickness	Y	GoogleNet, Breslow index	5	66.2	89.19	85	N/A	N/A
[165]	N	1	D	Y	Y	Y	Y	Classify lesion using fused features that extracted from deep learning and image processing	Y	ResNet50, Telangiectasia Vessel Detection Algorithm, transfer learning, GPU	5	N/A	N/A	N/A	N/A	N/A
[166]	Y	1	D	Y	Y	N	N	Classify skin lesions over IoT using deep learning	Y	CNN with nine layers, IoT,	7	81.4	N/A	N/A	N/A	N/A
[168]	Y	3	D	Y	Y	Y	N	Skin lesion classification using depthwise separable residual CNN	Y	Non-local means filter, contrast-limited adaptive histogram equalization, discrete Wavelet transforms, depthwise separable residual DCNN	2	99.5	99.31	100	N/A	N/A
		1	C	Y												
[169]	Y	1	D	Y	Y	Y	Y	Classify skin lesion using Attention Residual Learning	Y	Attention residual learning CNN, ResNet50, transfer learning, Augmentation, GPU	3	N/A	N/A	N/A	N/A	N/A
[170]	Y	1	D	Y	Y	N	N	Classify skin lesion using CNN with novel regularization method	Y	CNN 7 layers, standard deviation of the weight matrix, GPU	2	97.49	94.3	93.6	N/A	N/A
[171]	N	1	D	Y	N	Y	Y	Classify skin lesion by Incorporating the knowledge of dermatologists to CNN	Y	ResNet50, DermaKNet, Modulation Block, asymmetry block, AVG layer, Polar AVG layer.	3	91.7	N/A	65.2	N/A	N/A
[172]	Y	1	-	-	Y	Y	Y	Classify skin lesions based on the novel 7-point melanoma checklist using Multitask CNN	Y	Multitask CNN, 7-point melanoma checklist, Augmentation, GPU	3	87.1	77.3	89.4	63	N/A
[173]	Y	1	D	Y	Y	N	N	Classify skin lesion using Aggregated CNN	Y	ResNet50, ResNet101, fisher vector (FV), SVM, Chi-squared kernel, transfer learning, Augmentation, GPU	2	86.81	N/A	N/A	N/A	N/A
[174]	Y	-	-	-	Y	N	N	Using CNN as a feature extractor for skin lesion images and classify these features	Y	Alex-Net, ECOC SVM, transfer learning	4	94.2	97.83	90.74	N/A	N/A
[175]	Y	1	D	Y	Y	N	N	Classification of skin neoplasms using CNN and transfer learning with web and mobile application	N	Inception V3 (GoogleNet), transfer learning, Augmentation, GPU	-	91	N/A	N/A	N/A	N/A
[176]	N	1	C	N	Y	N	Y	An application used through the cloud to classify diseases of face skin	Y	LeNet-5, AlexNet and VGG16, transfer learning, Augmentation, GPU	5	N/A	N/A	N/A	N/A	N/A
[177]	Y	2	D	Y	Y	Y	Y	Skin lesion classification using 4 CNNs and ensembling of the final classification results	Y	AlexNet, VGG, ResNet-18, ResNet-101, SVM, MLP, random forest, transfer learning, Augmentation, GPU	3	87.7	85	73.29	N/A	N/A
[178]	Y	1	D	Y	Y	N	N	Compare the ability of deep learning model to classify skin lesions with expert dermatologists	Y	ResNet50, local outlier factor, transfer learning, GPU	2	N/A	87.5	60	N/A	N/A
[179]	N	1	D	N	Y	Y	Y	Evolving the deep learning model		PSO, hybrid learning PSO, Firefly Algorithm, spiral research action, probability distributions, crossover, mutation, K-Means, VGG16, Augmentation, GPU	2	73.76	N/A	N/A	N/A	N/A
		2	D	Y												
[181]	Y	3	D	Y	Y	Y	Y	Combine and expand current segmentation CNN to enhance the classification of skin lesions	Y	U-Net, ResNet34, LinkNet34, LinkNet152, fine-tuning, PyTorch, transfer learning, Augmentation, GPU, Jaccard-loss,	2	N/A	N/A	N/A	N/A	N/A
[182]	N	1	D	Y	N	Y	Y	skin lesion segmentation based using geodesic morphological active contour	Y	Gaussian filter, Otsu’s threshold, deformable models, partial differential equation, Mathematical morphology, active geodesic contour, neural network, deep learning, statistical region merging (SRM),	2	94.59	91.72	97.99	N/A	89
[183]	Y	1	-	-	Y	N	N	Erythema migrans and the other confounding lesions of skin using	Y	ResNet50, Keras, TensorFlow, fine-tuning, transfer learning, Augmentation, GPU	4	86.53	76.4	75.96	N/A	92.09
[184]	Y	1	D	Y	Y	N	N	Comparing between the CNN and 112 dermatologists for skin lesion detection	Y	ResNet50, fine-tuning, transfer learning, Augmentation, GPU	5	N/A	56.5	98.2	N/A	N/A
[185]	Y	2	D	Y	Y	Y	Y	Segmentation of skin lesions by ensemble the segmentation output of 2 CNN	Y	DEEPLABV3+, Mask R-CNN, ABCD, fine-tuning, transfer learning, Augmentation, GPU	2	94.08	89.93	95	N/A	N/A
[186]	Y	1	C	Y	N	Y	Y	Proposing an algorithm that able to train CNN with limited data	Y	Inception V3 (GoogleNet), PECK, SCIDOG, SVM, RF, fine-tuning, transfer learning, Augmentation, GPU	2	91	92	93	N/A	90.7
[187]	Y	1	C	N	Y	N	N	Classify skin disease of faces using Euclidean space to compute L-2 distance between images	Y	ResNet152, InceptionResNet-V2, fine-tuning, Euclidean space, L-2 distance, transfer learning, Augmentation, GPU	4	87.42	97.04	97.23	N/A	N/A
[188]	Y	1	D	Y	Y	Y	Y	Neural Architecture Search to increase the size of the network based on the dataset size	Y	VGG8, VGG11, VGG16, 5-fold validation, Neural Architecture Search (NAS), hill-climbing, transfer learning, Augmentation, GPU	2	77	N/A	N/A	N/A	N/A
[189]	Y	2	D	Y	Y	Y	Y	Skin lesion segmentation and pixel-wise classification using encoder and decoder network	Y	CNN, encoder-decoder deep network with skip connection, softmax, transfer learning, Augmentation, GPU	2	95	97	96	N/A	N/A
[190]	Y	2	D	Y	Y	Y	Y	Multitasks DCNN for skin lesion segmentation, detection, and classification	Y	Multitask DCNN, Jaccard distance, focal loss, Augmentation, GPU	2	95.9	83.1	98.6	N/A	95
[191]	Y	1	D	Y	Y	Y	Y	Light Lightweight CNN for skin lesion segmentation and classification	Y	Lightweight CNN, MobileNet, DenseNet, U-Net, focal loss, fine-tune, transfer learning, Augmentation, GPU	2	96.2	93.4	97.4	N/A	88.9
[192]	N	1	D	Y	Y	Y	N	Enhancement and classification of dermoscopic skin images	Y	StyleGANs, 43 CNNs (ResNet50, VGG11, VGG13, AlexNet, SENet, etc.) max voting, fine-tune, transfer learning, Augmentation, GPU	8	99.5	98.3	99.6	N/A	92.3
[193]	Y	1	D	Y	Y	Y	Y	Skin lesion classification based on CNN	Y	Deep-class CNN, Augmentation, GPU	2	75	73	78	N/A	N/A
[194]	Y	2	D	Y	Y	Y	Y	Skin lesion segmentation using encoder-decoder FCN	Y	FCN, GPU	3	96.92	96.88	95.31	N/A	N/A
[195]	Y	3	D	Y	Y	N	Y	Classify skin melanoma by extracting ROI and CNNS	Y	AlexNet, ResNet101, GoogleNet, Multiclass SVM, SoftMax, Histogram based windowing process, hierarchical clustering, fine-tune, transfer learning, Augmentation, GPU	3	98.14	97.27	98.60	N/A	88.64
		1	C	Y												
[196]	Y	3	D	Y	N	Y	Y	The fusion of extracted deep features of a skin lesion for classification	Y	Biorthogonal 2-D wavelet transform, Otsu algorithm, Alex and VGG-16, PCA, fusion, fine-tune, transfer learning, Augmentation, GPU	2	99.9	99.5	99.6	N/A	N/A
[197]	N	3	D	Y	Y	Y	Y	Ensemble multiscale and multi-CNN network	Y	EfficientNetB0, EfficientNetB1, SeResNeXt-50, fusion, fine-tune, transfer learning, Augmentation, GPU	7	96.3	N/A	N/A	91.3	82
[198]	Y	2	D	Y	Y	Y	Y	Classification of skin lesions by multiclass multilevel using traditional machine learning and transfer learning	Y	K-means, Otsu’s thresholding, GLCM, ANN. k-fold validation, AlexNet, fine-tune, transfer learning, Augmentation, GPU	4	93.02	87.87	98.17	97.96	N/A
[199]	N	2	D	Y	N	Y	Y	Optimized algorithm for weight selection to minimize the output of the network	Y	The bubble-net mechanism, Whale optimization algorithm (WOA), Lévy Flight Mechanism, genetic algorithm, shark smell optimization (SSO), world cup optimization algorithm, grasshopper optimization algorithm (GOA), particle swarm optimization algorithm (PSO), LeNet, fine-tune, transfer learning, GPU	2	N/A	N/A	N/A	N/A	N/A
[200]	Y	3	D	Y	N	Y	Y	semantic skin lesion segmentation with parameters reducing	Y	U-Net, FCN8s, DSNet, Augmentation, GPU	7	N/A	87.5	95.5	N/A	N/A
[201]	N	3	D	Y	Y	Y	Y	Different CNN network integration for segmentation and multiple classification stage	Y	Inception-v3, ResNet-50, Inception-ResNet-v2, and DenseNet-201	7	89.28	81	87.16	N/A	81.82
[202]	Y	3	D	Y	Y	Y	Y	Skin lesion segmentation based on CNN	Y	CIElab, FCN, U-Net, Augmentation, GPU	2	N/A	N/A	N/A	N/A	87.1
[205]	Y	1	D	Y	Y	N	N	Classify the challenging dataset ISIC2018	Y	AlexNet, 10-fold cross-validation, fine-tune, transfer learning, Augmentation, GPU	7	92.99	70.44	96	62.78	N/A
[204]	Y	1	D	Y	Y	N	N	Classify the challenging dataset ISIC2019	Y	GoogleNet, Similarity score, bootstrap weighted SVM classifier, SoftMax, fine-tune, transfer learning, Augmentation, GPU	9	98.70	95.6	99.27	95.06	N/A

## Data Availability

Not applicable.
